# Enterohepatic Recirculation-Mediated Reabsorption of Aristolochic Acid I: Revealed by Toxicokinetics and Metabolite Identification in Rats

**DOI:** 10.3390/toxics13110919

**Published:** 2025-10-27

**Authors:** Lieyan Huang, Lixing Nie, Xiao Ye, Zhi Lin, Ying Liu, Feng Wei

**Affiliations:** 1 National Institutes for Food and Drug Control, Chinese Academy of Medical Sciences and Peking Union Medical College, Beijing 100730, China; huanglyan2020@163.com; 2 National Institutes for Food and Drug Control, Beijing 102629, China; nielixing@163.com (L.N.); yexiao@nifdc.org.cn (X.Y.); linzhi@nifdc.org.cn (Z.L.); liuying@nifdc.org.cn (Y.L.); 3 The World Health Organization Collaborating Center for Herbal Medicine (CHN-139), Beijing 102629, China; 4 State Key Laboratory of Drug Regulatory Science, Beijing 102629, China

**Keywords:** aristolochic acid I, toxicokinetics, metabolism, enterohepatic recirculation

## Abstract

Aristolochic acid I (AAI) is widely recognized as a genotoxic and cytotoxic compound. To rationally propose detoxification strategies, it is essential to fully elucidate the in vivo disposition of AAI. Nevertheless, the toxicokinetic characteristics of AAI, particularly the possible involvement of the recirculation process, remain incompletely understood. In this research, toxicokinetics of AAI was studied following a single oral administration of AAI in Fisher rats (10, 30 and 100 mg/kg, *n* = 6). A method of ultra-performance liquid chromatography coupled with triple quadrupole tandem mass spectrometry (UPLC-QQQ-MS/MS) was developed to achieve the quantitation of AAI in rat plasma. Plasma concentration–time profiles and kinetic parameters were analyzed to characterize the toxicokinetic behavior of AAI. A secondary elevation was observed in the plasma concentration–time profiles of AAI, suggesting the existence of AAI reabsorption. The non-linear elimination kinetics of AAI might be attributed to capacity-limited excretion via bile. Additionally, the biliary excretion of AAI and several key metabolites was also explored through qualitative analysis of bile samples. For the first time, AAI*-O-*glucuronide was identified in bile, providing further support for enterohepatic recirculation (EHR)-mediated reabsorption of AAI. In conclusion, these findings provided solid evidence for EHR-mediated reabsorption of AAI in rats. The recirculation process might be a key mechanism responsible for the prolonged retention of AAI. In the future, detoxification strategies targeting the EHR process could be effective approaches to minimize the systemic exposure of AAI.

## 1. Introduction

Aristolochic acids (AAs), a group of nitrophenanthrene carboxylic acids, are naturally occurring phytochemicals found in various species of the Aristolochiaceae family, particularly within the *Aristolochia* and *Asarum* genera [1]. In the early 1990s, progressive interstitial nephritis was frequently reported in Belgian women who had ingested slimming pills containing AAs, which marked the first recognition of aristolochic acid nephropathy (AAN) [2]. Nephropathy cases related to the intake of AAs were also observed in Balkan regions, where wheat contaminated with seeds of *Aristolochia clematitis* L. was suspected to be responsible for the development of Balkan-endemic nephropathy [3]. Over the past few decades, AAN has been considered as a worldwide problem [4]. Although many countries have banned the prescription of AA-containing herbs, AA-induced toxic cases are still observed all over the world. In China, botanical remedies containing AAs remain officially listed in the Chinese Pharmacopoeia [5]. Additionally, AAs were proven to be persistent pollutants in the underground water and soil of many regions, resulting in continuous human exposure [3,6]. Apart from nephrotoxicity, AAs also exhibit carcinogenic properties. It has been proved by modern research that AAs are highly associated with the development of upper tract urothelial carcinoma and hepatocellular carcinoma [7,8]. The target organs of AAs extend beyond the kidney, also affecting the liver [9], upper urothelial tract [10] and bladder [11].

**Figure 1:**
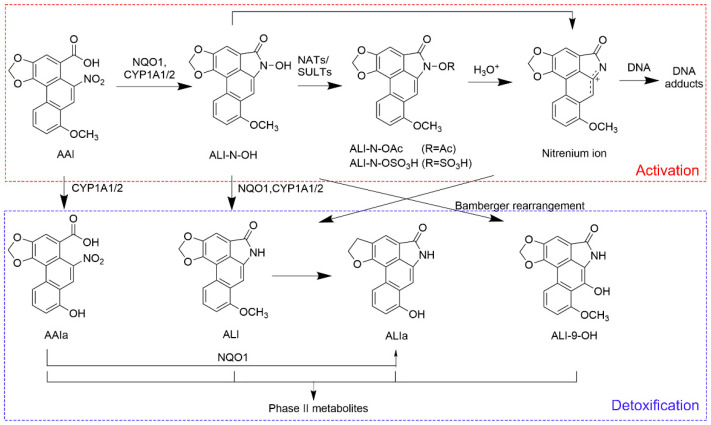
Proposed pathways of metabolic activation and detoxification of AAI. Arrows indicate enzymatic reactions, non-enzymatic processes and structure rearrangements during the generation of different metabolites. NATs: N-acetyltransferases; NQO1: quinone oxidoreductase 1; SULTs: sulfotransferases; ALI: aristolactam I; ALI-9-OH: 9-hydroxyaristolactam I; ALI-N-OAc: aristolactam I-N-acetoxy ester; ALI-N-OSO_3_H: aristolactam I-N-sulfate.

Over the years, extensive research has been conducted to uncover the toxic mechanisms of AAs. Aristolochic acid I (AAI) is one of the major toxic constituents of AAs and has been proven to exhibit a strong correlation with AAN and carcinoma development [12,13]. Research focusing on AAI has emerged as a representative and essential approach for understanding the toxicological mechanisms of AAs. The molecular mechanisms of AAI-induced nephrotoxicity encompass the induction of oxidative stress, DNA damage, mitochondrial dysfunction, cell fibrosis and cell apoptosis [14,15]. The proximal tubule of the kidneys is recognized as the major target of AAI, making tubular necrosis a typical pathological feature of AAN [14,15]. An article published in 2011 demonstrated that organic anion transporters 1 and 3 (OAT1/3) on the basolateral membrane of proximal tubular cells specifically transported AAI from the blood into the tubular epithelium, indicating the critical role of OATs in AAI-induced nephrotoxicity [16]. Regarding the carcinogenic property of AAI, the nitroreduction of AAI is identified as a crucial step during the formation of genotoxic metabolites. As depicted in Figure 1, AAI is catalyzed by NAD(P)H: quinone oxidoreductase 1 (NQO1) and microsomal cytochrome P450 1A1 and 1A2 (CYP1A1/2) to generate N-hydroxyaristolactam I (ALI-N-OH). The acetylation and sulfonation derivatives of ALI-N-OH readily form a reactive intermediate, namely, the cyclic nitrenium ion [17]. The electrophilic property of a nitrenium ion drives it to bind covalently to nucleophilic centers in DNA, forming mutagenic DNA adducts including 7-(deoxyadenosine-N6-yl)-aristolactam I (dA-ALI) and 7-(deoxyguanosine-N2-yl)-aristolactam I (dG-ALI) [18,19]. In AA-associated carcinogenesis, dA-ALI can induce A:T transversions on the non-transcribed strand, giving rise to the occurrence of an AA mutational fingerprint in oncogenes and tumor suppressor genes [20,21]. O-demethylation is regarded as a major in vivo detoxification pathway of AAI. The O-demethylated product of AAI, aristolochic acid Ia (AAIa), was observed to have less genotoxic potential and less nephrotoxicity [22].

Of particular concern, AAs maintain their toxic effects for prolonged periods post-administration. For instance, urothelial carcinoma was diagnosed in many patients 68 to 169 months after cessation of AA exposure [23]. Another investigation found that patients with high intake of AAs could develop progressive kidney failure even up to 1–7 years after exposure [24]. In 2021, an investigation explored the long-term hepatocarcinogenic effects of AAI in C3H/HeN suckling mice. Nine months after a single administration of AAI, pathological changes related to liver injury could still be observed in the livers of mice, with 78.57% of male mice and 41.67% of female mice developing liver cancer [25]. The biomarker of AAI exposure, dA-ALI, can be persistent decades after a distinct AAI exposure [26]. The strong covalent binding of AAI metabolites to nucleotides contributes to the persistent genotoxicity of AAI [18,19]. Therefore, efficient systemic clearance of AAI from exposed animals is crucial for minimizing its genotoxic effects. Before proposing detoxification strategies, it is essential to gain a deeper understanding of AAI kinetics—particularly its elimination characteristics. However, current knowledge regarding AAI elimination is limited and requires further investigation.

In several previous pharmacokinetic investigations of AAI, a “double-peak phenomenon” was observed in the plasma concentration–time profiles of AAI [27,28]. Nevertheless, in other investigations, the plasma concentration–time profiles of AAI presented the classic pattern of an initial increase to peak concentration followed by a gradual decline, without exhibiting a secondary elevation of plasma concentration [29,30]. Whether the occurrence of a double-peak phenomenon in AAI plasma concentration–time profile is an incidental observation or not requires rigorous experimental verification. Additionally, the underlying reason for the occurrence of a double-peak phenomenon in AAI plasma concentration–time profiles was not discussed in previous studies. The formation of multiple peaks in a plasma concentration–time profile is usually attributed to enterohepatic recirculation (EHR), delayed gastric emptying and discontinuous gastrointestinal absorption [31]. Investigating the potential recirculation of AAI would substantially improve current understanding of its pharmacokinetic behavior. This insight, in turn, would provide valuable guidance for proposing effective detoxification strategies for AAI.

This investigation was carried out on the basis of quantitative and qualitative analysis. The primary objective of this study was to comprehensively elucidate the toxicokinetic properties of AAI, with a particular focus on its reabsorption characteristics. Metabolites in bile samples were also analyzed to provide further evidence for the involvement of EHR during the in vivo disposition of AAI. Collectively, this work advances current understanding of AAI toxicokinetics and lays the groundwork for future research into its detoxification strategies.

## 2. Materials and Methods

### 2.1. Materials and Reagents

Reference standards of AAI (purity: 99.1%) and indomethacin (purity: 99.9%) were sourced from National Institutes for Food and Drug Control (Beijing, China). Formic acid, acetonitrile and methanol were of LC-MS grade and were purchased from Thermo Fisher Scientific (San Jose, CA, USA). Ammonium formate (purity: ≥99.0%) and sodium carboxymethyl cellulose (CMC-Na) were obtained from Sigma-Aldrich (St. Louis, MO, USA). Ultra-pure water was obtained from a Milli-Q system manufactured by Merck-Millipore (Overijse, Belgium).

### 2.2. Toxicokinetic Study

#### 2.2.1. Animals for Toxicokinetic Study

For toxicokinetic study, forty-eight 6-year-old F344 rats (specific pathogen-free grade) were enrolled in the study, with equal numbers of males and females. All rats were purchased from Charles River Laboratory (Raleigh, NC, USA). All rats were confirmed to be healthy based on a series of physical health examinations. Rats were acclimated for 5 days at a temperature of 20–26 °C, humidity of 40–70% and a 12 h light/dark circle. The rats were randomly divided into four groups (*n* = 12 per group), with equal numbers of males and females in each group. The allocation was performed separately for each sex (female and male) based on body weight using a stratified randomization method. During the experiment, rats were fasted and had access to water ad libitum. To minimize confounding factors, treatments and measurements were randomized within each session. Cages from different groups were evenly distributed across racks in the same room to reduce bias from light and temperature. This study was approved by the Institutional Animal Care and Use Committee (IACUC) of the National Drug Safety Evaluation Center (Approval No. IACUC-2024-K011, 20 September 2024).

During the experiment, each rat received a single oral administration via gastric gavage. Rats in the control group were administered with 0.5% sodium carboxymethyl cellulose (CMC-Na) suspension. For the treatment groups, rats were administered with gavage solution containing 10, 30 and 100 mg/kg of AAI. The gavage solution was prepared by uniformly dispersing the reference standard of AAI in the 0.5% CMC-Na solution. After oral administration, blood samples were collected from the postorbital vein plex of rats and immediately placed in EDTA tubes. To capture potential reabsorption which might occur long after AAI administration, 15 blood sampling time points were established for each dose group. A sparse sampling approach was employed to avoid excessive blood loss in each rat. For each dosage, 12 rats were subdivided into two groups. For the first group, blood samples were collected from 6 rats at 10, 30, 60, 90, 120, 240, 420, and 600 min after AAI administration; for the second group, blood samples were collected from these 6 rats at 12, 14, 16, 18, 20, 22, and 24 h after AAI administration. Therefore, the experimental design ensured that six biological replicates were obtained for each sampling time point, enabling robust toxicokinetic analysis.

#### 2.2.2. Plasma Sample Preparation

Rat plasma samples were prepared as follows: Blood was centrifuged at 13,200 rpm for 10 min. Then, 25 μL of retrieved plasma was transferred to 2 mL centrifuge tubes, followed by the addition of 75 μL of methanol/acetonitrile solution (*v*/*v*, 1∶1) containing indomethacin as internal standard (IS). After vortexing for 2 min and centrifugation for 10 min at 13,200 rpm, the supernatant was used as study samples for quantitative analysis. Quality control samples (QCs) and calibration standards were prepared by spiking blank plasma with different concentrations of AAI standard solution. The QCs and calibration standards were processed using identical procedures as the study samples.

#### 2.2.3. LC-MS/MS Parameters for Quantitation of AAI in Rat Plasma

A method of ultra-performance liquid chromatography coupled with triple quadrupole tandem mass spectrometry (UPLC-QQQ-MS/MS) was developed to achieve the quantitation of AAI in rat plasma. The LC-MS/MS analysis was performed using an Acquity UPLC I-Class system coupled with an Xevo TQ XS spectrometer (Waters, Milford, MA, USA). An Acquity UPLC BEH C18 column (2.1 mm × 50 mm, 1.7 μm, Waters, Milford, MA, USA) was employed for chromatographic separation. The mobile phases consisted of water containing 5 mmol/L ammonium formate/0.1% formic acid (A) and acetonitrile (B). The gradient elution was set as follows: 1–4 min, 10–90% B; 4–5 min, 90% B; 5–5.01 min, 90–10% B; 5.01–7 min, 10% B. The flow rate was set at 0.4 mL/min. The column temperature was maintained at 40 °C. A sample volume of 2 μL was injected for analysis.

Mass spectrometry detection was performed using a triple-quadrupole mass spectrometer equipped with an electrospray ionization source (Waters, Milford, MA, USA). The tandem mass spectrometry detection was carried out in positive ion mode with dynamic multiple reaction monitoring (MRM) acquisition. The ion source parameters were set as follows: capillary voltage, 0.5 kV; ion source temperature, 150 °C; desolvation gas temperature, 500 °C; cone gas flow rate, 150 L/h; nebulizer gas pressure, 7.0 bar; collision gas flow rate, 0.15 mL/min; desolvation gas flow rate, 1000 L/h. The optimized cone voltages for AAI and IS were 24 V and 32 V. The collision energies for AAI and IS were 10 eV and 22 eV. The ion transitions for quantification were *m*/*z* 359.0→298.0 for AAI and *m*/*z* 358.0 → 139.0 for IS.

#### 2.2.4. Method Validation for Quantitation of AAI in Rat Plasma

A full validation of the bioanalytical method for the quantitative analysis of AAI in rat plasma was performed in inherence to the ICH M10 guideline [32]. Selectivity, accuracy and precision, matrix effects, calibration curve and range, dilution integrity and stability were fully validated. The analyzed QCs included a lower limit of quantitation (LLOQ) sample, low QC (LQC), medium QC (MQC) and high QC (HQC). QCs of four concentration levels (LLOQ: 1 ng/mL; LQC: 3 ng/mL; MQC: 400 ng/mL; HQC: 800 ng/mL) were prepared by spiking AAI with blank plasma collected from 6 rats.

Selectivity was evaluated using blank plasma obtained from 6 rats. The responses attributable to interfering components were compared with the analyte response of the LLOQ sample. The response of interfering components in blank plasma should not be more than 20% of the AAI response and not more than 5% of the IS response in the LLOQ sample.

Accuracy and precision were evaluated by analyzing QCs within runs and between runs. For within-run evaluation, LLOQ, LQCs, MQCs and HQCs were analyzed in 5 replicates at each run. For between-run evaluation, 3 analytical runs were performed over three days and the obtained data was combined to calculated between-run results. The accuracy and precision should be within ±20% at the LLOQ and within 15% at the other concentration levels.

Matrix effect was evaluated using 3 replicates of LQCs and HQCs. The back-calculated concentrations of LQCs and HQCs should be within ±15% of the nominal concentration. The precision should be not be greater than 15%.

The calibration curve comprised a blank sample, a zero sample (blank plasma spiked with IS) and 9 concentration levels of calibration standards. A linear regression model was used to describe the concentration–response relationship. The deviation between the back-calculated concentration of each calibration standard and its nominal concentration should be within ±20% at the LLOQ and within 15% at the other concentration levels. The calibration range was defined within the LLOQ and the ULOQ.

For the evaluation of dilution integrity, calibration standard containing 5000 ng/mL of AAI was diluted 10-fold and 100-fold with blank plasma, respectively. The diluted samples were used as dilution QCs. In an analytical run, 5 replicates per dilution factor were tested to determine the accuracy and precision of diluted samples. The mean accuracy of the dilution QCs should be within ±15% of the nominal concentration and the precision should not exceed 15%.

Stability evaluation was carried out by analyzing LQCs and HQCs after storage under different conditions. Freeze–thaw stability was assessed after 3 cycles of freezing and thawing. Short-term stability was tested by keeping the LQCs and HQCs on the bench top at room temperature for the same duration as the study samples. Stability of the analyte in processed samples was tested by placing the processed samples in an autosampler for 16 h. Long-term stability was assessed by storing LQCs and HQCs at −20 °C for 15 days. The mean concentration at each QC level should be within ±15% of the nominal concentration.

#### 2.2.5. Data Analysis

A sparse sampling approach was employed in this research to avoid excessive blood loss in each rat. Pharmacokinetic data collected from different rats were pooled together using a naïve-pooling approach. The pooled dataset was imported into the Phoenix WinNonlin 8.3.5 software (Pharsight, Sunnyvale, CA, USA) and was subjected to non-compartmental analysis (NCA) to generate the following toxicokinetic parameters for AAI: area under the concentration–time curve from 0 to the last observation (AUC_0→t_), peak plasma concentration (C_max_), time to reach C_max_ (t_max_), apparent systemic clearance (CL/F), elimination half-life (t_1/2_) and apparent volume of distribution (V_d_). Prior to analysis, plasma concentrations below the LLOQ were set to zero. During the calculation of toxicokinetic parameters, no animals or data points were excluded during the experiment. Statistical comparisons of toxicokinetic parameters between different dose groups were conducted using GraphPad Prism 10.1.2 software (GraphPad Software, San Diego, CA, USA).

### 2.3. Bile Analysis

#### 2.3.1. Animals for Bile Analysis

Ten 6-week-old F344 rats were divided into a control group (*n* = 2) and a treatment group (*n* = 8), with equal numbers of males and females in each group. During the experiment, rats were fasted and had access to water ad libitum. Rats in the control group were administered with 0.5% CMC-Na suspension via gastric gavage. For the treatment group, rats were administered with 30 mg/kg of AAI by the same route. Rats underwent bile duct cannulation for bile sample collection. Bile samples were collected at consecutive intervals of 0–3 h, 3–6 h, 6–9 h and 9–12 h after administration.

#### 2.3.2. Bile Sample Preparation

For each bile sample, a 200 μL aliquot of bile was mixed with an equal volume of a methanol/acetonitrile (1:1, *v*/*v*) solution. After vortexing for 2 min, the mixture was centrifuged at 13,200 rpm for 10 min. The precipitate was discarded, and the supernatant was dried under nitrogen gas. The dried residue was reconstituted in 80 μL of methanol, vortexed for 2 min, and centrifuged at 13,200 rpm for 15 min. The resulting supernatant was used for further analysis.

#### 2.3.3. Qualitative Analysis of AAI Metabolites in Bile

Ultra-high-performance liquid chromatography (UHPLC) coupled with a Q-Exactive orbitrap mass spectrometer (Thermo Fisher Scientific, San Jose, CA, USA) was used to analyzed the metabolites of AAI in rat bile. An ACQUITY UPLC BEH C18 column (2.1 mm × 50 mm, 1.7 μm, Waters, Milford, MA, USA) was employed for chromatographic separation. The mobile phase consisted of water containing 5 mmol/L ammonium formate/0.1% formic acid (A) and acetonitrile (B). The gradient elution program was set as follows: 0–3 min, 5–10% B; 3–10 min, 10–23% B; 10–15 min, 23–50% B; 15–20 min, 50–95% B; 20–23 min, 95% B; 23–23.2 min, 95–5% B; 23.2–33 min, 5% B. For the analysis of all samples, the flow rate was set at 0.3 mL/min with the column temperature maintained at 40 °C, and the injection volume was 2 μL.

Metabolites in bile samples were analyzed using electrospray ionization (ESI) in both positive and negative ion modes. The spray voltage was set at 3500 V for positive mode and 3000 V for negative mode. The ion transfer tube temperature was maintained at 350 °C with a vaporizer temperature of 300 °C. The sheath gas and auxiliary gas flow rates were 35 Arb and 10 Arb. Full-scan MS data were acquired at a resolution of 35,000 within the mass range of *m*/*z* 100–1200. For MS/MS analysis, data-dependent acquisition (DDA) was performed at a resolution of 17,500 using high-energy collision dissociation (HCD). Post-acquisition data was imported into Compound Discoverer 2.1 software to identify AAI metabolites. Blank matrices were processed alongside samples to exclude the background interferences.

## 3. Results

### 3.1. LC-MS/MS Method Validation for the Detection of AAI in Rat Plasma

The calibration curve of AAI was linear within the concentration range of 1–1000 ng/mL (R2 = 0.996). The lower limit of quantification (LLOQ) of AAI in rat plasma was 1 ng/mL. The selectivity, accuracy and precision, matrix effect, dilution integrity and stability of the developed method were validated, with corresponding results listed in Table 1, Table 2, Table 3, Table 4 and Table 5. It was demonstrated by validation results that the developed method was suitable for quantifying AAI in rat plasma. Analysis of plasma samples was performed in compliance with the acceptance criteria established by ICH M10 guidelines [32], thereby ensuring the reliability of quantitation results.

### 3.2. Toxicokinetic Study of AAI in Rats

#### 3.2.1. Double-Peak Phenomenon of Plasma Concentration–Time Profiles

**Figure 2:**
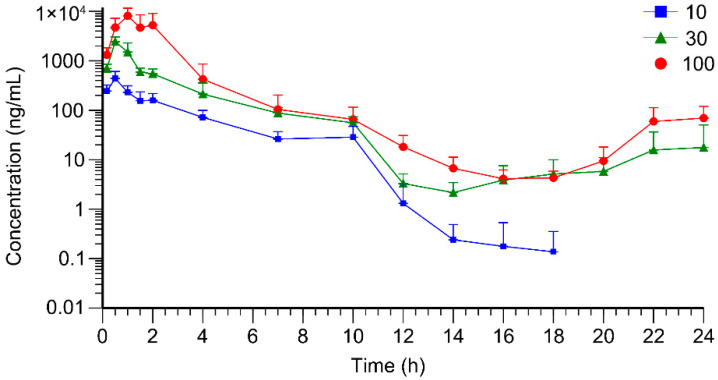
Average plasma concentration–time curves of AAI in rats (*n* = 6) after oral administration of AAI (10, 30, 100 mg/kg).

**Figure 3:**
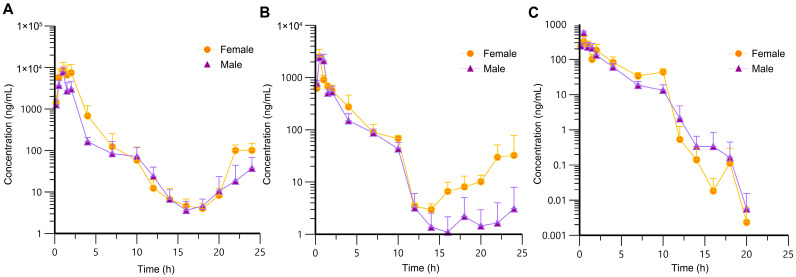
Average plasma concentration–time profiles of AAI in male and female rats following oral administration (*n* = 3 per sex). (**A**) High-dose group (100 mg/kg); (**B**) medium-dose group (30 mg/kg); (**C**) low-dose group (10 mg/kg).

The sparse sampling and naïve-pooling approaches are valid when there is no large variation among the studied subjects [33,34]. As an inbred strain, F344 rats selected for this study shared high genetic homogeneity and similar age, which permitted the adoption of the naïve-pooling approach. Within each dosage group, plasma concentrations generated from rats with the same sex and similar weight were pooled together to construct a plasma concentration–time profile, which encompassed the entire post-administration observation period. Raw data for the construction of toxicokinetic profiles are listed in Tables S1–S3 (Supplementary Materials). The average plasma concentration–time profiles of AAI are depicted in Figure 2 (*n* = 6). Following AAI administration, the plasma concentration of AAI rose to a peak and then gradually declined, which was consistent with the typical pharmacokinetic behavior. Notably, in the high- and medium-dose groups, a secondary elevation of plasma AAI concentration was observed at 14–24 h post-administration. This phenomenon indicated reabsorption of AAI into systemic circulation. For the low-dose group, AAI concentrations in many plasma samples were below LLOQ and were therefore assigned a fixed value of zero. Due to the limitations of the current analytical method, no distinct secondary peak was detectable in the low-dose group. As displayed in Figure 3, the plasma concentration–time profiles of AAI demonstrated sex-dependent differences across different dose levels. In high-dose and medium-dose groups, female rats exhibited higher systemic exposure compared to males, indicating a higher risk of AAI-related toxicity. Moreover, in these two groups, female rats exhibited a more pronounced secondary peak in AAI plasma concentration, suggesting enhanced recirculation of AAI in female rats. However, in the low-dose group, plasma AAI concentrations below LLOQ were assigned to zero, which precluded reliable evaluation of sex-dependent differences in recirculation.

#### 3.2.2. Non-Linear Elimination of AAI Indicated by Toxicokinetic Parameters

**Figure 4:**
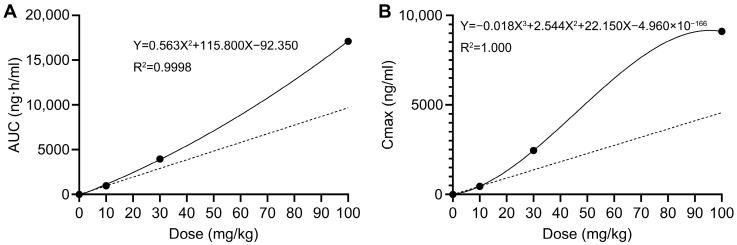
Average C_max_ and AUC_0→t_ values versus doses. The solid line represents the observed values, whereas the dashed line represents the assuming linear kinetics. (**A**) Average AUC_0→t_ values versus doses. (**B**) Average C_max_ values versus doses.

Naïve-pooled data generated via sparse sampling was subjected to non-compartmental analysis (NCA). Raw data for toxicokinetic calculation is provided in Table S4 (Supplementary Materials). The final results of toxicokinetic parameters are listed in Table 6. Toxicokinetic parameters between different dose groups were compared using one-way ANOVA. Among all parameters, only AUC and C_max_ showed significant differences across dose groups (*p* < 0.0001). As displayed in Figure 4, there was a superproportional relationship between the AUC and C_max_ with the escalating doses, suggesting the non-linear pharmacokinetics of AAI. A 3.33-fold increase in oral dose (from 30 mg/kg to 100 mg/kg) resulted in 3.7-fold (from 2453.15 to 9106.17 ng/mL) and 4.3-fold (from 3954.49 to 17,110.46 ng·h/mL) increase in C_max_ and AUC_0→t_ values, respectively. The greater-than-proportional increase in AUC_0→t_ and C_max_ with doses usually indicates non-linear elimination of drugs [13].

### 3.3. Identification of AAI Metabolites in Bile

#### 3.3.1. Detection of AAI-*O*-Glucuronide in Bile

**Figure 5:**
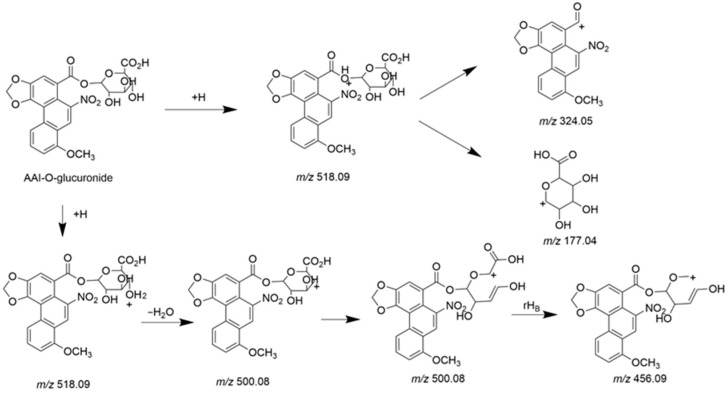
Chemical structure and proposed fragmentation pathways of AAI*-O-*glucuronide.

**Figure 6:**
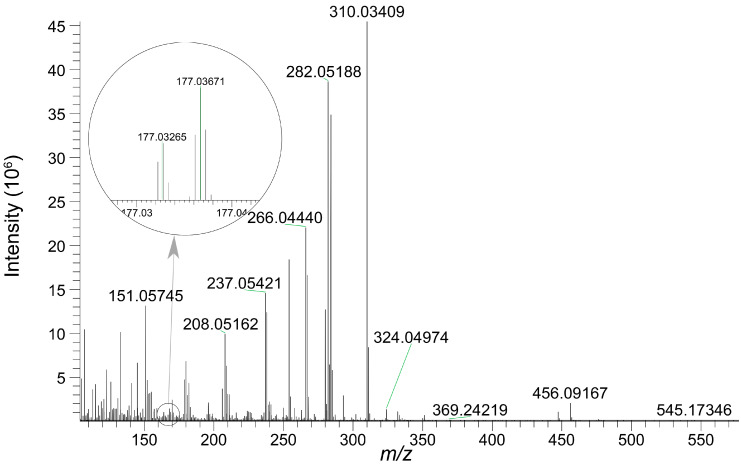
MS/MS spectrum of AAI*-O-*glucuronide eluted at 11.6 min.

Both AAI and its *O*-glucuronide were detected in bile, confirming the biliary excretion of AAI and its glucuronide. The parent compound, AAI, was detected in bile collected during 0–3 h and 3–6 h intervals post-administration. As shown in the extracted ion chromatogram (Figure S1), AAI was eluted at 16.60 min, sharing the same retention time as its reference standard. The MS/MS spectrum of the reference standard is displayed in Figure S1. Fragment ions observed at *m*/*z* 324.0504 and *m*/*z* 296.0674 were also reported in previous literatures as the characteristic ions of AAI [35]. The glucuronidation product of AAI, AAI*-O-*glucuronide, was detectable in bile collected during four consecutive periods after oral administration. The conjugation of a glucuronide moiety increased the polarity of the detected compound, resulting in an earlier elution time of AAI*-O-*glucuronide (11.6 min) compared to AAI. This research represents the first study to identify AAI*-O-*glucuronide in biological samples. The proposed chemical structure and fragmentation mechanism of AAI*-O-*glucuronide are illustrated in Figure 5. Identification of AAI*-O-*glucuronide was further supported by characteristic fragments observed in the MS/MS spectrum (Figure 6). The product ion at *m/z* 177.0367 was assigned to the protonated glucuronide moiety. Additionally, the fragment observed at *m/z* 324.0497 further confirmed that the -COOH group was the glucuronidation site of AAI.

#### 3.3.2. Detection of Genotoxic Intermediates in Bile

**Figure 7:**
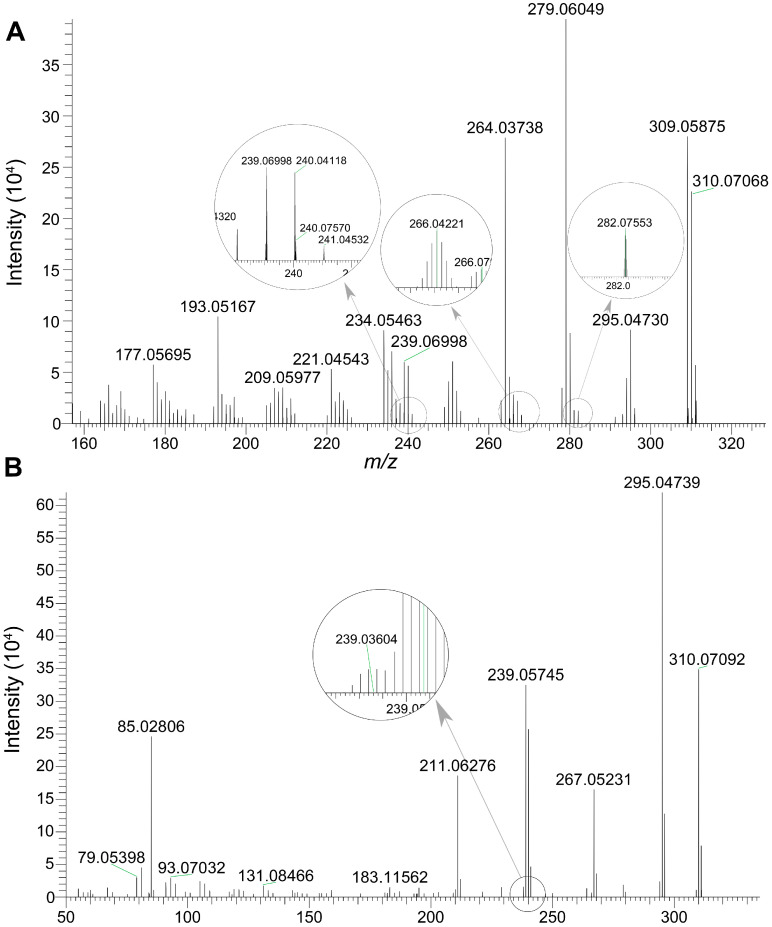
The MS/MS spectra of ALI-N-OH and ALI-9-OH. (**A**) MS/MS spectrum of ALI-9-OH eluted at 12.6 min. (**B**) MS/MS spectrum of ALI-N-OH eluted at 12.9 min.

The genotoxic intermediate, ALI-N-OH, together with its isomer, ALI-9-OH, were detected in bile samples. The chemical structures of two compounds are displayed in Figure 1. Fragmentation results of ALI-N-OH and ALI-9-OH are summarized in Table 7, and the corresponding MS/MS spectra are presented in Figure 7. According to the result of SwissADME calculation [36], ALI-9-OH exhibited a higher topological polar surface area (80.78 Å^2^) than ALI-N-OH (69.92 Å^2^), indicating a higher polarity of ALI-9-OH than ALI-N-OH. Therefore, during chromatographic separation using a reverse-phase column, ALI-9-OH should be eluted prior to ALI-N-OH. The peak eluted at 12.6 min was assigned to ALI-9-OH, while the peak eluted at 12.9 min corresponded to ALI-N-OH.

## 4. Discussion

For the first time, the recirculation process of AAI was confirmed by rigorous experiment in vivo. A double-peak phenomenon was observed in the plasma concentration–time profiles of AAI. In a prior study, the concentration of AAI in rat bile showed two distinct peaks after oral administration, supporting the potential involvement of EHR during the disposition of AAI [31,37]. Although delayed gastric emptying and discontinuous absorption might also contribute to the presence of multiple peaks in drug plasma profiles, secondary elevations caused by these two factors usually occur earlier after drug administration [38,39,40,41]. In contrast, in this experiment, the secondary peak was observed long after the oral administration of AAI. As a result, the EHR process of AAI was considered as the dominant reason leading to the secondary elevation of AAI plasma concentration during 14–24 h post-administration.

As was claimed in previous articles, drugs can undergo direct glucuronidation followed by biliary excretion and deglucuronidation in the intestine through enzymatic hydrolysis by gut bacteria. Therefore, the presence of a parent compound or its glucuronide conjugate in bile can serve as an indicator of potential EHR [42,43]. To qualitatively verify if the glucuronidation product of AAI could be excreted via bile, bile samples were collected from rats administered with AAI and were analyzed using a high-resolution mass spectrometer. Considering that metabolite concentration in the low-dose group might fall below the detection limit of the instrument, and that high-dose exposure might induce hepatotoxicity and disrupt bile excretion, a dose of 30 mg/kg was selected for the bile experiment. In this study, both AAI and its glucuronide conjugate were detected in bile. Biliary excretion of these two compounds provided further evidence for EHR-mediated reabsorption of AAI. The putative EHR process of AAI was described as follows: After oral administration and in vivo metabolism, AAI and its glucuronide conjugates were excreted into the intestine via bile. Then the glucuronide of AAI was deconjugated by microbial flora to release AAI aglycone. Given that AAI has a moderate passive diffusion ability across the Caco-2 monolayers [44], AAI in intestinal lumen could be reabsorbed into the systemic circulation by passive diffusion. Whether influx transporters participated during the intestinal uptake of AAI remained unknown. Ultimately, AAI re-entered the systemic recirculation and led to the secondary peak observed in the plasma concentration–time profile.

The existence of EHR was further substantiated by the non-linear kinetics of AAI. In this experiment, the dose gradient (10, 30 and 100 mg/kg AAI) was designed to evaluate potential dose-dependent kinetics which might arise from the saturation of transporters. As deciphered via toxicokinetic study, there were greater-than-proportional increases in both AUC and C_max_ with escalating doses, suggesting the non-linear elimination kinetics of AAI [17]. Such kinetics often arise from drug elimination processes that involve saturable transporters with definite capacities [45,46]. Since the biliary excretion of xenobiotics is typically mediated by active carrier transporters [47], the non-linear elimination kinetics of AAI might be attributed to its capacity-limited excretion via bile. Although the specific transporters mediating the biliary excretion of AAI are yet to be determined, saturation of these capacity-limited transporters was strongly implicated in the non-linear elimination kinetics of AAI.

Apart from the exploration of EHR-mediated reabsorption of AAI, this research also extended to the detection of important genotoxic intermediates of AAI in bile samples. The identification of ALI-N-OH in rat bile represented a critical finding, concerning its well-established genotoxic potential. It was claimed in an earlier investigation that dA-ALI and dG-ALI adducts were mainly formed through the Phase II activation of ALI-N-OH [48,49]. The acetylation and sulfation process of ALI-N-OH enhanced its electrophilic properties, contributing to a more efficient covalent binding of electrophilic cyclic nitrenium ions to DNA, and, ultimately, leading to an increased genotoxicity of AAI metabolites [17,48,49]. In this experiment, the detection of ALI-N-OH in bile offered new information for the exploration of AAI-associated hepatocellular carcinoma and biliary tract carcinoma [7,25,50]. The biliary excretion of ALI-N-OH not only confirmed the hepatic formation of a key genotoxic intermediate but also highlighted the biliary system as a critical site for AAI bioactivation and carcinogen exposure. As reported in a previous study, tumor mutation burden was significantly elevated in intrahepatic cholangiocarcinoma cases harboring the AA mutational signature compared to AA-unrelated patients, suggesting that AA exposure may induce DNA damage and promote the accumulation of somatic mutations in cholangiocarcinoma [50,51]. In this research, the detection of ALI-N-OH in bile provided a biochemical rationale for the development of AA-associated carcinoma. Future work could be undertaken to identify the transporters responsible for the uptake and efflux of AAI and its metabolites in epithelial cells lining the bile duct, offering critical insights into the toxicological study of AAI.

The study had several limitations that should be addressed in future research. In the present study, AAI was observed to exhibit non-linear elimination kinetics. While NCA was suitable for estimating parameters such as C_max_, t_max_ and AUC_0→t_, more sophisticated models are needed to obtain accurate values of t_1/2_ and CL/F under non-linear conditions. Methods such as non-linear mixed-effect modeling (NONMEM) could be adopted to characterize non-linear pharmacokinetics [52,53]. Furthermore, conventional modeling methods are insufficient to accurately characterize multi-phasic plasma concentration profiles. To address this limitation, several advanced pharmacokinetic modeling strategies have been developed, including the time-delay model, multicompartment model and oscillatory pharmacokinetic model [54,55,56,57]. Given both the non-linear kinetics and multiple-peak profiles, future studies should seek to provide a more advanced modeling method to estimate the t_1/2_ and CL/F of AAI. For the identification of compounds of interest in bile, only two rats were included in the control group. While bile samples from two control rats were sufficient to serve as negative controls for excluding background interference during the qualitative analysis of AAI and its metabolites, the limited sample size restricted the assessment of interindividual or sex-dependent variability. Therefore, future investigations on the biliary excretion of AAI and its metabolites should be conducted with an expanded cohort of animals to ensure the robustness of conclusions. Moreover, the EHR process of AAI remains to be further verified using different approaches. In the future, it is recommended to employ a linked-rat model to directly monitor the EHR of AAI [58,59,60]. Meanwhile, the specific transporters responsible for the intestinal absorption and reabsorption of AAI remain to be fully elucidated. By unmasking the uptake transporters of AAI, novel detoxification strategies could be proposed to interrupt the EHR process of AAI and thereby minimize its systemic retention and cumulative toxicity.

## 5. Conclusions

Based on a toxicokinetic study and metabolite identification, this research revealed EHR-mediated reabsorption of AAI in rats, bringing new perspectives for the in vivo disposition of AAI. The occurrence of EHR was indicated by the characteristic double-peak phenomenon observed in the plasma concentration–time profiles of AAI. Non-linear elimination kinetics of AAI were clearly demonstrated by the disproportionate increase in C_max_ and AUC values with escalating doses (10, 30 and 100 mg/kg). The non-linear elimination kinetics of AAI might be attributed to saturation of hepatic transporters involved in the biliary excretion of AAI. The presence of AAI and its glucuronide in bile provided further support for the existence of the EHR process. These findings provide new insights on the detoxification strategies of AAI. Future studies should be carried out to unveil the specific transporters responsible for the biliary excretion and intestinal uptake of AAI. Moreover, interventions aiming at disrupting the EHR process may emerge as an effective detoxification approach to minimizing the cumulative toxicity of AAI caused by recirculation.

## Figures and Tables

**Table 1:** Selectivity of AAI in rat plasma samples.

Samples	AAI		IS	
	Retention Time (min)	Peak Area	Retention Time (min)	Peak Area
S1	2.6	40.51	2.9	79.33
S2	/	/	2.9	59.95
S3	2.61	22.06	2.96	13.43
S4	2.61	16.56	2.9	125.17
S5	2.59	89.64	2.9	384.43
S6	2.6	72.89	2.9	394.33
LLOQ sample	2.6	690.57	2.9	15,681.9

**Table 2:** Accuracy and precision of AAI in rat plasma samples (*n* = 5).

	Nominal Conc. (ng/mL)	Mean Calculated Conc. (ng/mL)	Accuracy (% Dev)	Precision (% CV)
Between-run	1	1.01	0.80	10.25
	3	2.81	−6.44	8.17
	400	389.67	−2.58	3.65
	800	762.48	−4.69	4.34
Within-run	1	1.03	2.54	8.91
	3	2.77	−7.53	6.03
	400	387.25	3.19	2.13
	800	749.75	−6.28	2.74

**Table 3:** Stability of AAI in rat plasma samples (*n* = 3).

Stability	Nominal Conc. (ng/mL)	Mean Calculated Conc. (ng/mL)	Accuracy (%Dev)	Precision (%CV)
Freeze–thaw	3	3.12	4.10	3.71
	800	810.42	1.30	2.79
Short-term	3	3.10	3.46	3.43
	800	776.07	−2.99	3.28
Post-processing	3	2.68	−10.53	2.91
	800	705.33	−11.83	1.94
Long-term	3	3.20	6.54	2.58
	800	837.66	4.71	8.33

**Table 4:** Evaluation of matrix effects of AAI in rat plasma samples (*n* = 3).

Matrix Source	Nominal Conc. (ng/mL)	Mean Calculated Conc. (ng/mL)	Accuracy (%Dev)	Precision (%CV)
1	3	3.03	1.04	1.64
	800	712.88	−10.89	2.89
2	3	2.85	−4.87	3.59
	800	694.71	−13.16	4.26
3	3	2.98	−0.64	3.81
	800	683.17	−14.60	2.87
4	3	3.02	0.80	1.32
	800	751.34	−6.08	1.21
5	3	2.95	−1.54	0.99
	800	714.51	−10.69	2.41
6	3	2.83	−5.72	3.70
	800	729.29	−8.84	1.41

**Table 5:** Evaluation of dilution integrity of AAI in rat plasma samples (*n* = 5).

Sample	Dilution Factor	Nominal Conc. (ng/mL)	Mean Calculated Conc. (ng/mL)	Accuracy (%Dev)	Precision (%CV)
1	10	500	450.54	−9.89	0.04
2	100	50	52.60	5.20	0.07

**Table 6:** Toxicokinetic parameters of AAI in rat plasma.

Parameters	Units	Dosage		
		10 mg/kg	30 mg/kg	100 mg/kg
C_max_	ng/mL	457.36 ± 128.76	2453.15 ± 560.61	9106.17 ± 2177.55
t_max_	h	0.69 ± 0.60	0.58 ± 0.17	1.17 ± 0.37
t_1/2_	h	1.31 ± 0.51	2.79 ± 0.74	3.01 ± 0.45
V_d_	L	3.36 ± 1.97	4.67 ± 0.81	8.42 ± 7.24
CL/F	L/h	1.66 ± 0.37	1.22 ± 0.30	1.13 ± 0.59
AUC_0→t_	ng·h/mL	968.66 ± 139.79	3954.49 ± 542.83	17,110.46 ± 7555.68

Data are presented as mean ± SD.

**Table 7:** Fragments of ALI-N-OH and ALI-9-OH.

Retention Time (min)	Precursor Ion (*m*/*z*)	Fragment Ion (*m*/*z*)	Identification Result
12.6	310.0707	295.0473 [M+H-·CH_3_]^+^	ALI-9-OH
		282.0755 [M+H-CO]^+^	
		266.0422 [M+H-CO-·OCH_3_]^+^	
		264.0626 [M+H-CO-H_2_O]^+^	
		240.0757 [M+H-CO-·N=C=O]^+^	
		239.0700 [M+H-CO-HN=C=O]^+^	
12.9	310.0709	295.0474 [M+H-·CH_3_]^+^	ALI-N-OH
		267.0523 [M+H-·CH_3_-CO]^+^	
		239.0360 [M+H-·CH_3_-CO-H_2_N≡ C·]^+^	

## Data Availability

The raw data supporting the conclusions of this article will be made available by the authors on request.

## Supplementary Materials

The following supporting information can be downloaded at https://www.mdpi.com/article/10.3390/toxics13110919/s1: Figure S1: The extracted ion chromatograms and MS/MS spectrum of AAI. Table S1: AAI plasma concentration of rats in low-dose group (*n* = 6 for each time point). Table S2: AAI plasma concentration of rats in medium-dose group (*n* = 6 for each time point). Table S3: AAI plasma concentration of rats in high-dose group (*n* = 6 for each time point). Table S4: Toxicokinetic parameters generated from pooled dataset. File S1: ARRIVE Checklist of animal experiments.

## Abbreviations

The following abbreviations are used in this manuscript:

AA	Aristolochic acid
AAN	Aristolochic acid nephropathy
ALI	Aristolactam I
ALI-9-OH	9-Hydroxyaristolactam I
ALI-N-OAc	Aristolactam I-N-acetoxy ester
ALI-N-OH	N-hydroxyaristolactam I
ALI-N-OSO_3_H	Aristolactam I-N-sulfate
AUC	Area under the curve
AUC_0→t_	Area under the concentration–time curve from 0 to the last observation
CL	Clearance
C_max_	Maximum concentration
CMC-Na	Sodium carboxymethyl cellulose
CYP1A1/2	Microsomal cytochrome P450 1A1 and 1A2
dA-ALI	7-(Deoxyadenosine-N6-yl)-aristolactam I
dG-ALI	7-(Deoxyguanosine-N2-yl)-aristolactam I
EHR	Enterohepatic recirculation
F344	Fisher 344
HQC	High-quality control
IACUC	Institutional Animal Care and Use Committee
ICH	M10 guideline
IS	Internal standard
LC-MS/MS	Liquid chromatography coupled with tandem mass spectrometry
LLOQ	Lower limit of quantification
LQC	Low-quality control
MQC	Medium-quality control
MRM	Multiple reaction monitoring
NAT	N-acetyltransferase
NCA	Non-compartmental analysis
NONMEM	Non-linear mixed-effect modeling
NQO1	NAD(P)H: quinone oxidoreductase 1
OAT	Organic anion transporter
QC	Quality control
SULT	Sulfotransferase
t_1/2_	Half-life time
t_max_	Time to maximum concentration
UHPLC	Ultra-high-performance liquid chromatography coupled with triple-quadrupole tandem mass spectrometry
ULOQ	Upper limit of quantification
UPLC-QQQ-MS/MS	Ultra-performance liquid chromatography coupled with triple-quadrupole tandem mass spectrometry
V_d_	Apparent volume of distribution
