# Supplementary material for: Enterohepatic Recirculation-Mediated Reabsorption of Aristolochic Acid I: Revealed by Toxicokinetics and Metabolite Identification in Rats
Source: Toxics. 2025 Oct 27;13(11):919. doi: 10.3390/toxics13110919 (PMC12656546; doi:10.3390/toxics13110919)
Supplement: Supplementary file 1 [file toxics-13-00919-s001.zip › 20251008 Supplementary materials.pdf]

Supplementary Materials

Enterohepatic Recirculation-Mediated Reabsorption of Aristolochic Acid I: Revealed by Toxicokinetics and Metabolite Identification in Rats

Lieyan Huang [1,2,†], Lixing Nie [2,3,4,†], Xiao Ye [2], Zhi Lin [2], Ying Liu [2] and Feng Wei [2,3,4,*]

[1] National Institutes for Food and Drug Control, Chinese Academy of Medical Sciences and Peking Union Medical College, Beijing 100730, China; huanglyan2020@163.com
[2] National Institutes for Food and Drug Control, Beijing 102629, China; nielixing@163.com (L.N.); yexiao@nifdc.org.cn (X.Y.); linzhi@nifdc.org.cn (Z.L.); liuying@nifdc.org.cn (Y.L.)
[3] The World Health Organization Collaborating Center for Herbal Medicine (CHN-139), Beijing 102629, China
[4] State Key Laboratory of Drug Regulatory Science, Beijing 102629, China
* Correspondence: weifeng@nifdc.org.cn; Tel.: +86-10-5385-2020
† These authors contributed equally to this work.

Supplementary Figures

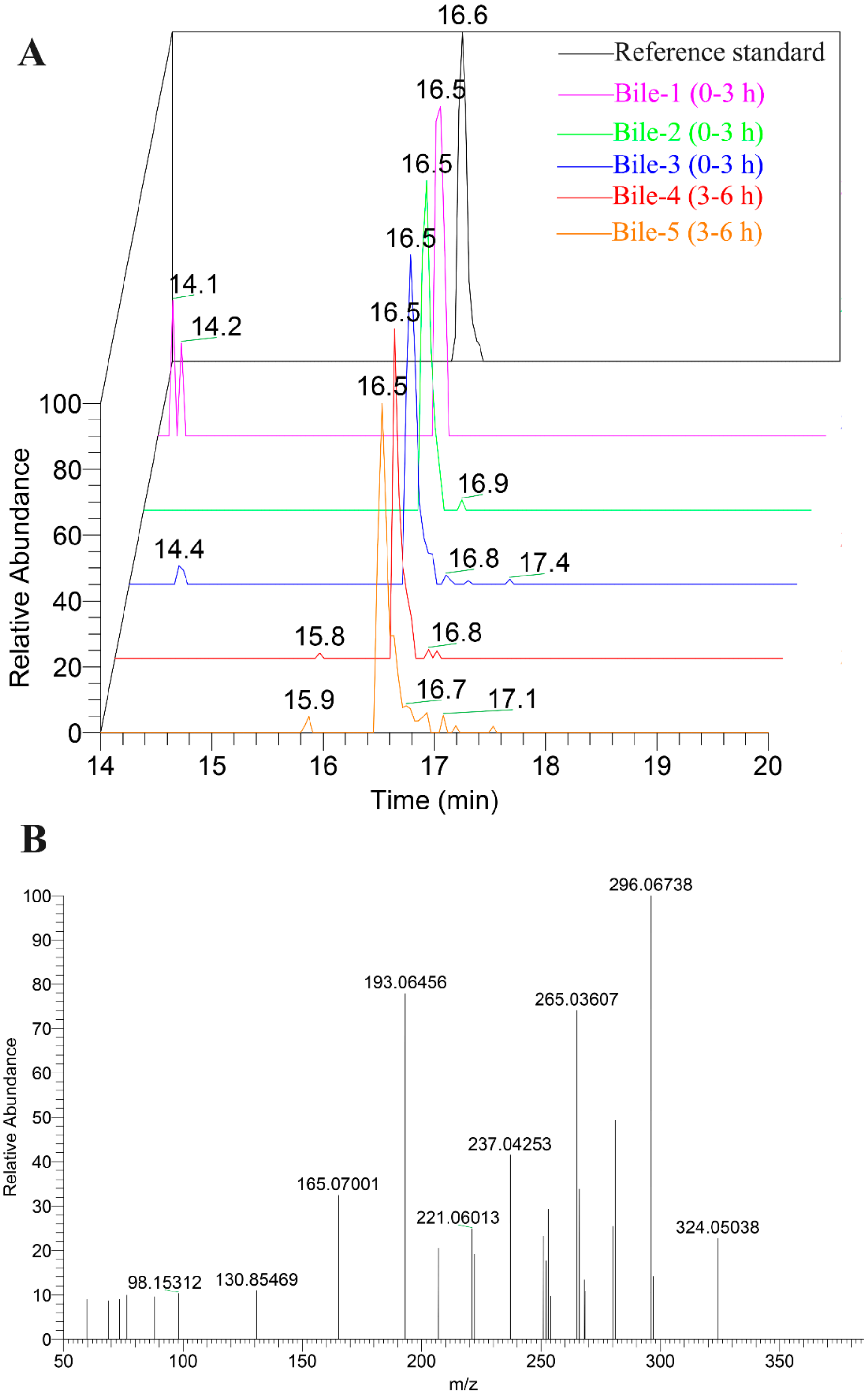

Figure S1. The extracted ion chromatograms and MS/MS spectrum of AAI. (A) Extracted ion chromatograms of AAI in standard solution and bile samples. (B) MS/MS spectrum of AAI generated from chromatographic peak eluted at 16.6 min.

Supplementary Tables

Table S1 AAI plasma concentration of rats in low-dose group (n=6 for each time point).

Group	Time points (h)	AAI plasma concentration (ng/mL)					
		M101 (group 1), M104 (group 2)	M102 (group 1), M105 (group 2)	M103 (group 1), M106 (group 2)	F101 (group 1), F104 (group 2)	F102 (group 1), F105 (group 2)	F103 (group 1), F106 (group 2)
1	0.167	302.65	205.66	224.48	361.73	237.84	131.58
	0.5	640.11	496.76	561.81	348.84	161.56	436.18
	1	320.59	171.91	184.15	218.72	153.69	342.08
	1.5	295.58	175.49	161.16	122.35	58.12	123.57
	2	135.36	134.41	131.07	93.20	247.55	212.86
	4	45.53	79.65	58.36	82.38	47.04	118.25
	7	24.53	14.80	16.15	40.40	23.83	38.02
	10	18.34	7.28	14.52	56.42	36.96	37.99
2	12	1.11	5.17	0.00	0.00	0.00	1.35
	14	0.00	0.00	0.00	0.00	0.00	0.00
	16	0.00	0.00	0.00	0.00	0.00	0.00
	18	0.00	0.00	0.00	0.00	0.00	0.00
	20	0.00	0.00	0.00	0.00	0.00	0.00
	22	0.00	0.00	0.00	0.00	0.00	0.00
	24	0.00	0.00	0.00	0.00	0.00	0.00

Plasma concentrations below the LLOQ of quantitation method were set to a fixed value of zero.

Table S2 AAI plasma concentration of rats in medium-dose group (n=6 for each time point).

Group	Time points (h)	AAI plasma concentration (ng/mL)					
		M201 (group 1), M204 (group 2)	M202 (group 1), M205 (group 2)	M203 (group 1), M206 (group 2)	F201 (group 1), F204 (group 2)	F202 (group 1), F205 (group 2)	F203 (group 1), F206 (group 2)
1	0.167	677.97	891.85	775.45	694.87	747.04	424.53
	0.5	2114.14	2496.11	2604.27	3133.29	2919.69	1424.98
	1	1312.37	2360.84	2630.67	814.02	1128.36	777.08
	1.5	413.26	508.51	594.78	649.03	685.24	712.70
	2	634.01	354.12	602.07	438.37	724.22	539.10
	4	90.61	161.84	196.08	93.85	264.87	468.11
	7	72.74	83.75	104.71	69.29	131.52	64.74
	10	26.96	52.18	50.62	70.78	76.59	58.20
2	12	1.92	6.45	1.24	2.81	3.93	3.49
	14	0.76	2.74	0.61	3.87	2.17	2.87
	16	0.61	2.32	0.38	4.36	5.23	10.35
	18	0.83	5.49	0.29	6.28	4.31	13.62
	20	0.91	3.14	0.30	7.63	13.88	9.00
	22	0.40	4.38	0.15	6.11	48.38	35.23
	24	0.23	8.71	0.34	6.62	84.22	6.41

Table S3 AAI plasma concentration of rats in high-dose group (n=6 for each time point).

Group	Time points (h)	AAI plasma concentration (ng/mL)					
		M301 (group 1), M304 (group 2)	M302 (group 1), M305 (group 2)	M303 (group 1), M306 (group 2)	F301 (group 1), F304 (group 2)	F302 (group 1), F305 (group 2)	F303 (group 1), F306 (group 2)
1	0.167	1009.67	1062.01	1721.81	1913.03	1643.16	607.21
	0.5	2999.47	3184.21	4967.56	1741.22	7104.04	8362.47
	1	6490.96	6245.89	10563.14	3115.53	9980.23	12351.60
	1.5	1664.97	2038.97	4522.83	3778.52	3945.25	12165.24
	2	1799.39	2407.42	4896.69	9005.21	2601.40	10921.55
	4	136.32	213.45	139.82	336.71	1280.18	446.76
	7	173.06	63.95	15.64	63.08	33.78	275.77
	10	122.68	64.66	30.04	8.77	38.31	129.34
2	12	16.05	14.02	42.26	19.30	13.11	4.59
	14	2.18	6.90	11.25	12.80	5.22	1.93
	16	5.46	4.41	1.05	3.74	3.08	7.06
	18	7.04	3.67	3.01	4.52	4.90	2.70
	20	25.70	2.27	3.98	11.14	9.92	3.92
	22	47.98	1.65	5.50	142.21	79.80	80.57
	24	58.08	2.38	52.27	136.79	46.66	121.57

Table S4 Toxicokinetic parameters generated from pooled dataset.

Dosage	Animals	C_{max} (ng/mL)	t_{max} (h)	$t_{1/2}$ (h)	Vd (L)	CL/F (L/h)	$AUC_{0\to t}$ (ng·h/mL)
Low-Dose	M101, M104	361.73	0.17	0.61	1.21	1.38	990.96
	M102, M105	247.55	2.00	1.21	2.67	1.53	824.62
	M103, M106	436.18	0.50	1.00	1.53	1.06	1221.57
	F101, F104	640.11	0.50	1.15	3.03	1.82	1055.94
	F102, F105	496.76	0.50	1.66	4.74	1.98	876.13

	F103, F106	561.81	0.50	2.23	6.96	2.17	842.71
Medium-dose	M201, M204	3133.29	0.50	3.08	5.23	1.18	3446.36
	M202, M205	2919.69	0.50	3.85	4.46	0.80	4703.09
	M203, M206	1424.98	0.50	3.33	4.94	1.03	3783.95
	F201, F204	2114.14	0.50	2.02	5.21	1.79	3228.59
	F202, F205	2496.11	0.50	2.73	5.21	1.32	3983.95
	F203, F206	2630.67	1.00	1.71	2.97	1.20	4581.00
High-dose	M301, M304	9005.21	2.00	20.86	23.01	0.76	17484.11
	M302, M305	9980.23	1.00	3.11	3.33	0.74	17260.39
	M303, M306	12351.60	1.00	2.58	1.48	0.40	32135.80
	F301, F304	6490.96	1.00	3.83	11.28	2.04	9262.46
	F302, F305	6245.89	1.00	2.80	7.23	1.79	9698.81
	F303, F306	10563.14	1.00	2.72	4.20	1.07	16821.21
